# Mixture of *Rhodiola rosea* and *Nelumbo nucifera* Extracts Ameliorates Sleep Quality of Adults with Sleep Disturbance

**DOI:** 10.3390/nu16121867

**Published:** 2024-06-14

**Authors:** Yunna Kim, Won Kyung Lee, Hyein Jeong, Hyuck Jai Choi, Mi-Kyung Lee, Seung-Hun Cho

**Affiliations:** 1 College of Korean Medicine, Kyung Hee University, Seoul 02447, Republic of Korea; yunna.anna.kim@khu.ac.kr; 2 Kyung Hee University Medical Center, Kyung Hee University, Seoul 02447, Republic of Korea; 3 Research Group of Neuroscience, East-West Medical Research Institute, WHO Collaborating Center, Kyung Hee University, Seoul 02447, Republic of Korea; 4 LG H&H R&D Campus, Daejeon 34114, Republic of Korea; wksophie@lghnh.com; 5 LG Science Park in Magok, LG H&H Co., Ltd., Seoul 07795, Republic of Korea; jhippo@lghnh.com; 6 Korean Society for Pharmacoepidemiology and Risk Management, Seoul 16499, Republic of Korea; nicchoi@naver.com

**Keywords:** sleep disturbance, *Rhodiola rosea*, *Nelumbo nucifera*, natural product, dietary supplement, ISI

## Abstract

Chronic sleep disturbance affects daily functioning, leading to decreased concentration, fatigue, and higher healthcare costs. Traditional insomnia medications are often associated with adverse side effects. This study investigated the efficacy of a novel compound derived from *Rhodiola rosea* and *Nelumbo nucifera* extracts (named RNE) in improving sleep quality with fewer side effects. The study included individuals between the ages of 20 and 65 with subthreshold insomnia and evaluated the effects of RNE on sleep, fatigue, and quality of life. Participants took 750 mg of RNE daily at bed-time for two weeks. The study used the Insomnia Severity Index (ISI), the Pittsburgh Sleep Quality Index (PSQI), a sleep diary, the Fatigue Severity Scale (FSS), and the Short Form 36 Health Survey (SF-36) for assessments. Of the 20 participants, 13 completed the study and showed significant improvements in sleep quality. The results showed improvements in ISI and PSQI scores, a 57% reduction in wake-time after sleep onset, and improved sleep efficiency. Although FSS scores remained unchanged, significant improvements were seen in SF-36 physical and mental health scores. The results suggest that RNE is an effective, low-risk option for sleep disturbance, significantly improving sleep quality and overall wellbeing without significant side effects.

## 1. Introduction

Sleep disturbance is a common symptom, but chronic exposure results in a suboptimal health status [1]. Chronic sleep disturbances can result in the low performance of activities during the day, including decreased concentration and lethargy, leading to a poor quality of life and an increased burden of medical use and costs. As sleep is crucial for health, sleep deficits are associated with various diseases. Sleep deficiency is an indicator or a risk factor for neurodegenerative diseases such as Alzheimer’s disease and Parkinson’s disease [2,3,4]. In addition, sleep difficulty has a bidirectional relationship with psychiatric diseases such as depression and anxiety [5,6]. The association between sleep disturbance and other health conditions, including obesity, pain, and male infertility, has been investigated [7,8,9].

In the United States, 16.3% of adults diagnosed with sleep disorders and 12.7% of adults with sleep problems reported using sleep aids [10]. Currently, a pharmacological approach accounts for the most considerable portion of insomnia treatments, with most of the drugs, including sedatives or hypnotics, being synthetic chemicals [11,12]. Consequently, the market for sleeping pills has grown; the dependence on sleeping pills has also risen. Zolpidem, the most commonly used medication for insomnia, has been recognized for its fast action, but it can cause side effects such as increased respiratory arousal, decreased attention, hallucinations, and suicidality, posing problems for both patients and society [13]. Therefore, there is a need for new pharmacological approaches such as using natural resources, which are generally considered to have fewer side effects, and the demand for sleep-enhancing supplements is increasing.

*Rhodiola rosea* Linn., commonly referred to as roseroot, has been approved by the Korean Food and Drug Administration as a supplement to improve stress-induced fatigue. Rosavin, a compound found in *R. rosea*, was reported to have potential therapeutic benefits for stress-related conditions and depression [14]. The study examined 3% rosavin and 1% salidroside combinations in animal models, showing anti-depressant, adaptogenic, and anxiolytic effects by reducing immobility and resting times while increasing active behaviors [15]. The combination reduced CRF-induced anorexia by lowering postsynaptic serotonergic receptor stimulation, indicating anti-anorexia and anti-stress effects [16]. Combined with eleutherosides, schisandrins, salidroside, and tyrosol, rosavin enhanced stress tolerance in BALB/c mice [17]. *Nelumbo nucifera* Gaertn., also known as lotus, is a medicinal herb and its seeds have been traditionally used in East Asia to treat people suffering from sleep disturbances. Neferine, a key phytochemical extracted from the seeds of *N. nucifera*, has demonstrated neuroprotective effects, including sleep induction [18]. It synergizes with thiopental to enhance sleep and exhibits sedative and anxiolytic effects similar to diazepam without affecting motor coordination [19]. Neferine also shows anti-depressant-like effects through serotonin receptor interactions [20]. Its anxiolytic/sedative effect has been confirmed in an animal model, suggesting that the seeds could be effective in treating sleep disorders caused by anxiety or depression [21].

This study investigated an extract prepared using a mixture of *R. rosea* roots and *N. nucifera* seeds extract (named RNE) as a possible new agent that could improve sleep quality without the side effects found in other insomnia medications. We examined changes in sleep quality, fatigue, and quality of life after RNE administration in adults with subthreshold insomnia.

## 2. Materials and Methods

This clinical trial was designed as a pre-post study. The study was ethically and scientifically conducted in accordance with the Korean Good Clinical Practice and the Helsinki Declaration. The study protocol was approved by the Kyung Hee University Korean Medical Hospital Institute Review Board (KOMCIRB-170320-HR-009). The study was performed from 27 June 2017 to 26 September 2017. All participants were provided explanations about the study and enrolled after voluntarily providing written informed consent.

### 2.1. Participants

We recruited adults aged between 20 and 65 years with subthreshold sleep disturbances. A sample size of 20 was planned as it was a pilot study. We included participants who voluntarily consented to participate and signed the informed consent form. We screened participants and collected clinical information from those who met the following inclusion criteria: (1) males or females aged 20 to 65 years old; (2) normal daily life, but with objective or subjective sleeping problems; (3) an Insomnia Severity Index (ISI) score of 8–14; (4) volunteering to participate in the clinical trial and agreeing to give informed consent. The exclusion criteria were individuals who (1) had been clinically diagnosed as having sleep disorders such as sleep apnea syndrome or restless leg syndrome; (2) were under treatment for alcohol/substance abuse or psychosis; (3) were unable to sustain a regular sleep schedule due to irregular work times, shifts, or night duties; (4) had a past history of an allergic reaction after the administration of herbal medicine; (5) were pregnant or lactating females or females at risk of becoming pregnant due to inappropriate contraception; (6) had participated in other clinical trials examining different medication within the previous month; (7) were expected to miss the scheduled study visits due to trips abroad or any expected long-term absence during the trial period; (8) were currently taking medication or supplements for sleep; (9) had a habit of consuming more than the standard recommendation of alcohol more than three times a week; (10) had a habit of drinking more than three cups of coffee a day; (11) had an abnormal laboratory finding; and (12) were deemed inappropriate for the clinical trial by the researchers.

### 2.2. Intervention

The raw materials of ethanol extract of *R. rosea* and ethanol extract of *N. nucifera* were purchased from Hyundai Bioland (Ansan, Republic of Korea). We standardized *R. rosea* and *N. nucifera* with their active substances, rosavin and neferine, respectively. The investigational product was prepared as capsules containing an extract (375 mg) prepared using the roots of *R. rosea* and seeds of *N. nucifera* at a 2:1 ratio by weight. The investigational products were encapsulated with the mixture in capsules at the Good Manufacturing Practices certified facility of Suheung Co., Ltd. (Cheongju, Republic of Korea). The participants were asked to orally ingest 2 capsules/day (750 mg/day) at bed-time for 2 weeks. If compliance was less than 70%, the participant was eliminated from the study.

### 2.3. Outcome Measures

The outcomes were measured at the baseline and 1 and 2 weeks after RNE administration. The primary outcomes were the differences in ISI and Pittsburgh Sleep Quality Index (PSQI) scores, while the secondary outcomes were the differences in the sleep diary, Fatigue Severity Scale (FSS), and Short Form 36 Health Survey (SF-36) scores.

#### 2.3.1. Insomnia Severity Index (ISI)

The ISI is a subjective self-report measurement tool that comprises a 5-point scale, with 7 items assessing the severity of sleep onset, sleep maintenance, early morning awakening problems, sleep dissatisfaction, interference of sleep difficulties with day-time functioning, noticeability of sleep problems by others, and related distress [22]. A score of 8–14 is classified as subthreshold insomnia.

#### 2.3.2. Pittsburgh Sleep Quality Index (PSQI)

The PSQI, developed by Buysse, is an efficient tool to measure the quality of sleep and sleep disturbance [23]. This tool uses a self-report questionnaire comprising 19 questions and 7 components (component 1: subjective sleep quality; 2: sleep latency; 3: sleep duration; 4: habitual sleep efficiency (SE); 5: sleep disturbance; 6: use of sleeping medication; and 7: day-time dysfunction). Each component includes related questions, with scores ranging from 0 to 3. A total score > 5 indicates poor sleep.

#### 2.3.3. Sleep Diary

A sleep diary was used to examine the overall aspect of sleep. Sleep-onset latency (SOL), wake-time after sleep onset (WASO), total sleep time (TST), and SE were calculated; sleep and day-time satisfaction data (e.g., day-time exercise, caffeine use, and alcohol use) were also collected. The sleep diary, containing records of sleep patterns since the last visit, was analyzed. The sleep diaries of weeks 1 and 2 were compared using the average SOL, WASO, TST, and SE scores during that week.

#### 2.3.4. Fatigue Severity Scale (FSS)

The FSS assesses day-time fatigue caused by insomnia. This tool is a commonly used self-report questionnaire that evaluates self-perceived feelings related to fatigue [24]. A higher score indicates higher fatigue.

#### 2.3.5. Short Form (36) Health Survey (SF-36)

The SF-36 was used to assess changes in the quality of life and diverse aspects of function [25]. Eight aspects were evaluated: physical functioning (PF), role limitations due to physical problems (RP), bodily pain (BP), general health (GH), vitality (VT), social functioning (SF), role limitations due to emotional problems (RE), and mental health (MH). Changes in the score of each aspect were compared. A higher score indicated a better quality of life.

### 2.4. Safety

The participants visited every week and were checked for vital signs, compliance with the intervention, and adverse events. Blood urea nitrogen, creatinine, aspartate transaminase (AST), and alanine aminotransferase (ALT) levels were estimated at the last visit.

### 2.5. Statistical Analysis

An intent-to-treat (ITT) analysis was used; if not applicable, a per-protocol analysis was used instead. Missing data were assumed using the last observation carried forward (LOCF) method for the ITT analysis. As the sample size was too small to achieve normality, the Friedman test and the Wilcoxon signed-rank test were used, if applicable. A statistical significance was set at *p* < 0.05 and a 95% confidence interval. Analyses were performed using IBM SPSS Statistics 22 (IBM Corp., Armonk, NY, USA).

## 3. Results

### 3.1. Participants

In total, 6 males (30%) and 14 females (70%) were included in this study. The average age was 33.40 ± 14.27 years. Of the participants. 12 (60%) were social drinkers, 4 (20%) smoked, and 15 (75%) drank approximately 1 cup of coffee a day on an average (Table 1).

The sleep statuses of the participants at screening are shown in Table 1. The average ISI score was 12.85 ± 1.39. Each participant had difficulties initiating sleep and the average frequency was 4.63 ± 1.79 days/week. Twelve participants (60%) had difficulties maintaining sleep and the average frequency was 4.67 ± 2.76 days/week. Although 11 individuals (55%) woke early in the morning, with an average frequency of 2.92 ± 2.22 days/week, 17 (85.0%) felt sleepy in the day-time, averaging 4.75 ± 2.01 days/week. Some experienced day-time discomfort due to sleep disturbances, including fatigue, sleepiness, eye strain, headache, grogginess, and memory decline. One participant took a sleeping pill, three used a sleep aid or supplement, and two had used cognitive behavioral therapy in the past. None of the participants included in the study were under any treatment a month before the trial or had a history of alcohol or substance abuse.

In all, 13 participants completed the study. Six individuals withdrew their consent to participate and one dropped out because of low compliance.

### 3.2. Sleep

**Figure 1:**
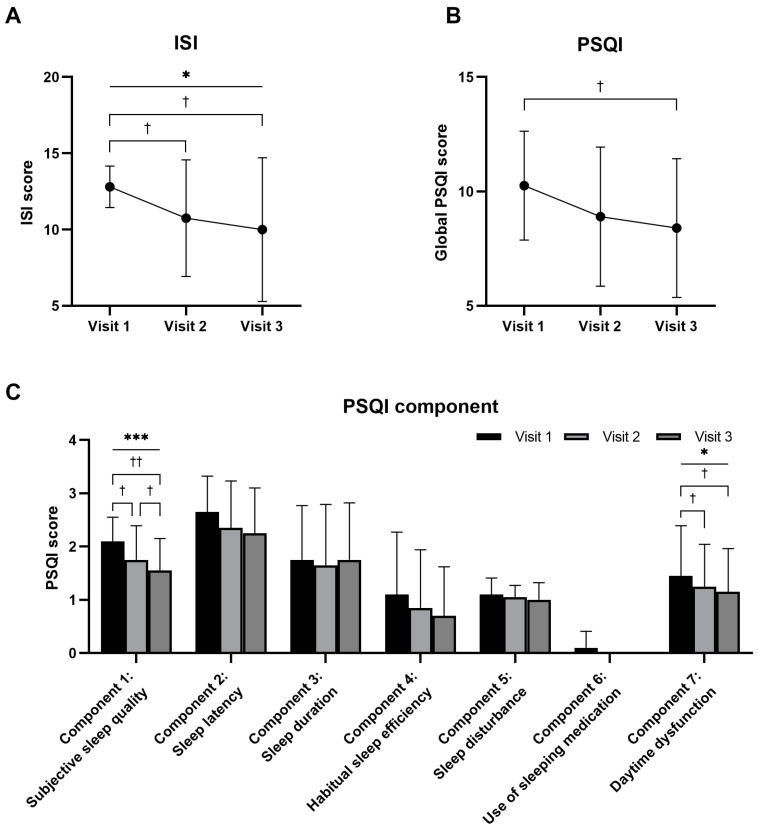
Comparison of Insomnia Severity Index (ISI) (**A**) and Pittsburgh Sleep Quality Index (PSQI) (**B**,**C**) scores for every visit after oral administration of a mixture of *Rhodiola rosea* and *Nelumbo nucifera* extracts (named RNE) (n = 20). Notes: Intent-to-treat analysis. Statistical significance evaluated using the Friedman test: * *p* < 0.05; *** *p* < 0.001. Statistical significance evaluated using the Wilcoxon signed-rank test: † *p* < 0.05; †† *p* < 0.01.

The ISI scores for visits 1, 2, and 3 were 12.80 ± 1.36, 10.75 ± 3.82, and 10.00 ± 4.71, respectively. Based on Friedman’s test, these changes were significant over time (*p* = 0.0180). Statistically significant differences were also observed between visits 1 and 2, and visits 1 and 3 (visit 1 vs. visit 2, *p* = 0.0299; visit 2 vs. visit 3, *p* = 0.2009; visit 1 vs. visit 3, *p* = 0.0191) (Figure 1A).

The PSQI scores for visits 1, 2, and 3 were 10.25 ± 2.38, 8.90 ± 3.04, and 8.40 ± 3.03, respectively. Although the change over time was not statistically significant (*p* = 0.0675), there was a statistically significant difference between visits 1 and 3 (visit 1 vs. visit 2, *p* = 0.0547; visit 2 vs. visit 3, *p* = 0.1878; visit 1 vs. visit 3, *p* = 0.0209) (Figure 1B). Components 1 (subjective sleep quality) and 7 (day-time dysfunction) also showed significant differences over time. In contrast, components 2 (sleep latency), 3 (sleep duration), 4 (habitual SE), 5 (sleep disturbance), and 6 (use of sleeping medication) did not show statistically significant differences between the three visits (Figure 1C).

**Figure 2:**
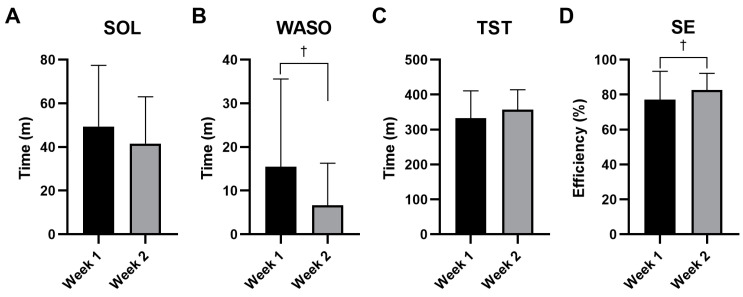
Comparison of sleep diary data for each week after oral administration of a mixture of *Rhodiola rosea* and *Nelumbo nucifera* extracts (named RNE) (n = 13). Sleep-onset latency (SOL) (**A**), wake-time after sleep onset (WASO) (**B**), total sleep time (TST) (**C**), sleep efficiency (SE) (**D**) were analyzed. Notes: Per-protocol analysis. Statistical significance evaluated using the Wilcoxon signed-rank test: † *p* < 0.05. Abbreviations: SOL: sleep-onset latency; WASO: wake-time after sleep onset; TST: total sleep time; SE: sleep efficiency.

As the sleep diary measured the sleep status of weeks 1 and 2, it was difficult to obtain the baseline values. Therefore, we were unable to implement LOCF for the participants who dropped out after visit 1 and a per-protocol analysis was performed. The SOL, which was 49.37 ± 28.08 min for week 1, decreased to 41.53 ± 21.51 min for week 2; however, the difference was not statistically significant (*p* = 0.2213) (Figure 2A). In contrast, WASO was 15.47 ± 20.09 min for week 1 and statistically significantly decreased to 6.68 ± 9.60 min for week 2 (*p* = 0.0159) (Figure 2B). TST was 332.47 ± 78.08 min for week 1; this increased to 357.45 ± 56.51 min for week 2, but the increase was not statistically significant (*p* = 0.2787) (Figure 2C). SE was 77.05 ± 16.34% for week 1 and significantly increased to 82.65 ± 9.55% for week 2 (*p* = 0.0392) (Figure 2D).

### 3.3. Fatigue

The FSS assesses fatigue during the day-time; the higher the score, the greater the fatigue. The FSS scores for visits 1, 2, and 3 were 37.65 ± 8.67, 38.50 ± 9.24, and 37.15 ± 7.29, respectively. The change in FSS total score over time was not statistically significant (*p* = 0.2757) and there were no statistically significant differences between the visits (visit 1 vs. 2, *p* = 0.5747; visit 2 vs. 3, *p* = 0.1546; visit 1 vs. 3, *p* = 0.7264) (Table 2).

### 3.4. Quality of Life

**Figure 3:**
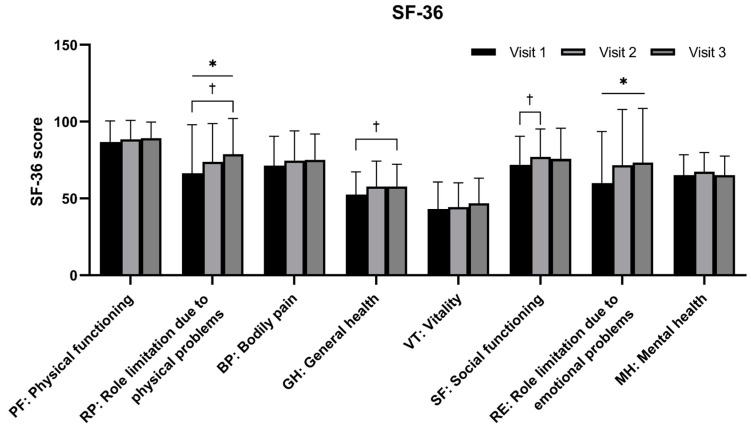
Comparison of Short Form (36) Health Survey (SF-36) scores for every visit after oral administration of a mixture of *Rhodiola rosea* and *Nelumbo nucifera* extracts (named RNE) (n = 20). Notes: Intent-to-treat analysis. Statistical significance evaluated using the Friedman test: * *p* < 0.05. Statistical significance evaluated using the Wilcoxon signed-rank test: † *p* < 0.05.

RP and RE showed statistically significant changes in scores over time (*p* = 0.0361 and 0.0457, respectively). Although the score change over time was not statistically significant, significant changes were seen in GH between visits 1 and 3 (*p* = 0.0296) and in SF between visits 1 and 2 (*p* = 0.0461). There were no statistically significant differences in PF, BP, VT, or MH between the three visits (Figure 3).

### 3.5. Change in Sleep According to Age Group

**Figure 4:**
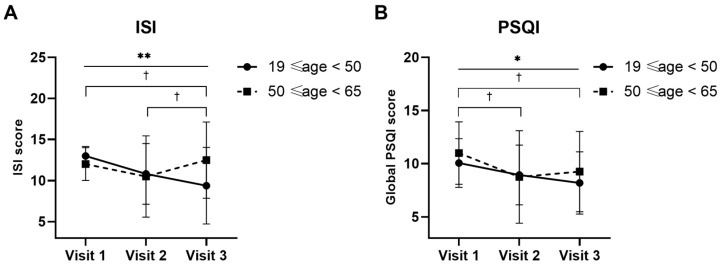
Comparison of Insomnia Severity Index (ISI) (**A**) and Pittsburgh Sleep Quality Index (PSQI) (**B**) scores for every visit after oral administration of a mixture of *Rhodiola rosea* and *Nelumbo nucifera* extracts (named RNE) in two age groups, 19 ≤ age < 50 (n = 16) and 50 ≤ age < 65 (n = 4). Notes: Intent-to-treat analysis. Statistical significance evaluated using the Friedman test and is indicated as follows: * *p* < 0.05; ** *p* < 0.01 in the age group of 19 ≤ age < 50. Statistical significance also evaluated using the Wilcoxon signed-rank test and is indicated as follows: † *p* < 0.05 in the age group of 19 ≤ age < 50.

The ISI and PSQI scores were analyzed according to age to observe the effectiveness of treatment on sleep, with the aim of excluding the effect of age. The ISI scores for the age group 19 ≤ age < 50 for visits 1, 2, and 3 were 13.00 ± 1.16, 10.81 ± 3.69, and 9.38 ± 4.66, respectively. The results of Friedman’s test indicated that these changes were statistically significant over time (*p* = 0.0015). Additionally, statistically significant differences were also observed between visits 2 and 3, and visits 1 and 3 (visit 1 vs. visit 2, *p* = 0.0522; visit 2 vs. visit 3, *p* = 0.0171; visit 1 vs. visit 3, *p* = 0.0125) (Figure 4A). The ISI scores for participants aged 50 to 65 for visits 1, 2, and 3 were 12.00 ± 2.00, 10.50 ± 4.93, and 12.50 ± 4.66, respectively. There was no significant difference between visits (Friedman’s test, *p* = 0.2921; Wilcoxon signed-rank test, visit 1 vs. visit 2, *p* = 0.5930; visit 2 vs. visit 3, *p* = 0.1088; visit 1 vs. visit 3, *p* = 1.0000) (Figure 4A).

The PSQI scores for the age group 19 ≤ age < 50 for visits 1, 2, and 3 were 10.06 ± 2.29, 8.94 ± 2.82, and 8.19 ± 2.93, respectively. The change over time was statistically significant (*p* = 0.0419). There was a statistically significant difference between visits 1 and 2, and visits 1 and 3 (visit 1 vs. visit 2, *p* = 0.0474; visit 2 vs. visit 3, *p* = 0.1064; visit 1 vs. visit 3, *p* = 0.0216). The PSQI scores for the age group 50 ≤ age < 65 for visits 1, 2, and 3 were 11.00 ± 2.94, 8.75 ± 4.35, and 9.25 ± 3.78, respectively. There was no statistically significant difference between visits and the score for visit 3 slightly increased, similar to the ISI results (Friedman’s test, *p* = 0.0675; Wilcoxon signed-rank test, visit 1 vs. visit 2, *p* = 0.4142; visit 2 vs. visit 3, *p* = 0.1573; visit 1 vs. visit 3, *p* = 0.5292) (Figure 4B).

### 3.6. Adverse Events

The overall compliance was 72.66 ± 40.16%. Adverse events with a possible causal relationship to the intervention included constipation (n = 2) and a dry mouth (n = 1). No clinically significant changes were observed in the other safety variables, including vital signs and laboratory test results.

## 4. Discussion

The current study evaluated the efficacy and safety of RNE in adults with subthreshold sleep disturbances. The subjective and objective sleep status improved after two weeks of administration and no serious adverse events were encountered. However, although RNE could enhance the physical and emotional levels related to the quality of life, it did not significantly relieve fatigue.

This study showed that RNE has the potential to improve overall sleep quality. Subjective evaluation indices for sleep, including the ISI score, PSQI total score, and component 1, improved over time. In the per-protocol analysis, participants who adhered to the trial almost recovered to a normal range of sleep parameters (see Tables S1–S4, which depict the sociodemographic characteristics and sleep status as well as the various scores evaluated after RNE administration under the per-protocol analysis). The ISI score decreased from 12.69 to 8.15, approaching the 8-point cut-off value of the ISI. The PSQI decreased from 10.62 to 7.46, close to a normal score of 5 points (Table S2). It was also noted that SE, the indicator of the objective parameters for sleep quality, significantly improved, as documented in the SE of the sleep diary and component 4 of the PSQI. The SE of the sleep diary was 82.65% in the second week, which is close to 85% and the normal range of sleep. Considering the definitions of ‘>85%’ (0 points) and ‘75–84%’ (1 point) in component 4 of the PSQI, the decrease from 1.31 to 0.54 was consistent with the SE in the sleep diary. Considering the characteristics of sleep that the participants could confront every night, the analysis of those who fully completed the trial should be referred to subsidiarily.

Both the subjective and objective outcome measures of sleep were alleviated after the administration of RNE. The increased subjective sleep quality and SE indicate that RNE probably positively affects sleep disturbance. Along with the subjective quality, the time parameters of sleep also improved. In the sleep diary, SOL decreased by 8 min, WASO decreased by 9 min, and TST increased by 25 min. A similar pattern was observed in the corresponding items in components 2 and 3 of the PSQI. As it is speculated that subjective sleep quality is related to slow-wave sleep and sleep maintenance [26], satisfaction with sleep could also be attributed to a reduction in WASO. This reduction was comparable with other treatment research, where cognitive behavioral therapy decreased SOL and WASO from 60–70 min to 35 min, and melatonin reduced SOL by 7.2 min [27].

RNE contributed to improvements in GH and sleep but did not change fatigue, as revealed by the FSS and VT scores in the SF-36. However, changes in component 7 of the PSQI and other items in the SF-36 reflected an improvement in day-time symptoms. Although the feeling of fatigue or energy loss persisted, there was an overall improvement in both physical and mental factors. A reduction in RE and MH showed that RNE could ameliorate mental aspects such as depression and anxiety. In Korea, *R. rosea* has been approved as a functional health food with the health claim ‘may help relieve fatigue by stress’ [28]. The seeds of *N. nucifera* have been traditionally used as anxiolytics in Korean medicine and their effects have been examined in previous studies [19,29]. Although PF did not show a significant difference, its absolute values were the highest among the SF-36 items. Considering that PF is known to have a high ceiling effect [30,31], the physical factors represented by PF, RP, and GH generally increased. This implies that RNE could assist physical recovery. Nevertheless, the relationship between the recovery of physical factors and improved sleep quality from RNE needs to be further explored.

The analysis of the ISI and PSQI scores according to age groups was conducted to observe the effectiveness of the treatment on sleep whilst excluding the potential confounding effect of age. For the younger age group (19 ≤ age < 50), the ISI scores showed a significant improvement over time. The PSQI scores also showed a significant improvement, which did not significantly change over time in the results of total participants. Conversely, for the older age group (50 ≤ age < 65), both the ISI and PSQI scores did not show a significant change between visits. These findings indicate that RNE could significantly improve both insomnia severity and sleep quality in younger adults (19 ≤ age < 50), but might not have a significant effect in older adults (50 ≤ age < 65). These results highlight the importance of considering age when evaluating the efficacy of sleep treatments and suggest that younger individuals may respond more favorably to RNE supplementation to improve sleep-related issues. Analyzing the sleep duration of 730,187 participants revealed a significant difference in sleep patterns at the ages of 33 and 53 years [32]. SOL increases between the late teens and 20s, remains consistent from age 30 to around 50 years, and then steadily increases after age 50. WASO shows a consistent 10 min increase per decade from ages 30 to 60, with minimal changes observed beyond 60 years. In contrast to other sleep parameters, which tend to stabilize after the age of 60, sleep efficiency continues to gradually decline with advancing age [33]. Nevertheless, due to the limited number of elderly participants, further research with larger sample sizes in the older age group may be necessary to fully comprehend the potential advantages of RNE for different age demographics.

Constipation and dry mouth were expected adverse events. The causal relationships need to be considered: one case of constipation improved after menstruation during the period of administration and the case of dry mouth resolved before the intake period ended. Therefore, it may be considered that no serious adverse events were observed during this study.

RNE both reduced the latency to sleep and increased sleep duration; its sleep-promoting, anxiolytic, and anti-convulsant effects were confirmed using mice with pentobarbital-induced sleep. Its plausible mechanism is its impact upon GABAergic and serotonergic systems. The 2:1 ratio of RNE was determined by comparing the efficacy of each ratio between *R. rosea* and *N. nucifera* on pentobarbital-induced sleep in mice [34]. The administration of 250 mg/kg *R. rosea* and *N. nucifera* single extracts improved sleep duration by 10.1% and 13.1%, respectively, compared with a saline-treated control group. A mixture of these extracts at ratios of 1:1, 2:1, and 4:1 at 250 mg/kg showed greater improvements of 14.6%, 16%, and 15.2%, respectively. For SOL, at a concentration of 500 mg/kg, single extracts of *R. rosea* and *N. nucifera* showed improvements of 19.3% and 16.8%, respectively. In comparison, a mixture of these extracts at ratios of 1:1, 2:1, and 4:1 at 500 mg/kg showed greater improvements of 22.5%, 26.7%, and 24.8%, respectively. Of these samples, the 2:1 ratio showed the most significant improvements in both sleep duration and sleep-onset latency [34]. The mechanism of RNE was studied in a preclinical setting. The administration of RNE to ICR mice for six days did not result in a statistically significant increase in melatonin levels, although there was a tendency for melatonin levels to rise with an increase in a dose-dependent manner. In contrast, serum serotonin levels significantly increased at doses of 250, 500, and 1000 mg/kg compared with the control group. Furthermore, the binding of antagonists to GABA, serotonin 2A, and serotonin 2C receptors was inhibited, indicating the potential mechanisms through which these extracts exert their effects [34]. These results were similar to those of previous studies. *R. rosea* itself has been reported to improve sleep-related parameters such as latency to sleep onset and sleep duration in animals, protecting them from learning and memory deficits due to sleep deprivation [35,36,37]. Its mechanism on its protective effect on sleep is suggested to result from changes in serotonergic and GABAergic immune-related mechanisms, providing protection from oxidative stress and neuron injury [35]. *N. nucifera* has been documented to increase nonrapid eye movement sleep and change subjective night-time activity, sleep bouts, and sleep time by regulating GABAergic receptors [38,39]. Procyanidin B2 and neferine, a compound from *N. nucifera*, improve sleep latency and sleep duration [40]. *R. rosea* also improved depression and anxiety and demonstrated an enhanced resistance to stress in clinical trials [41,42,43,44], while *N. nucifera* was also reported to have anti-depressant and anxiolytic effects in animal models. Both these herbs may alleviate sleep disturbances derived from depressive or anxiety disorders [19,21,45].

Efforts to search for effective and safe sleep aids from natural products continue. The effects of *Valeriana officinalis* (valerian), *Piper methysticum* (kava), *Matricaria recutita* (German chamomile), *Passiflora Incarnata* (passionflower), *Hypericum perforation* (St. John’s Wort), and *Panax ginseng* (Korea Red Ginseng) have been reported in clinical studies and animal experiments to date [46]. *Valeriana officinalis* (valerian) improved sleep in adults diagnosed with insomnia and middle-aged adults with mild sleep complaints in various clinical trials [47,48,49,50]. Although the sleep-promoting effects of these various natural products have been reported, with the exception of a few plants, their effects were insignificant or their underlying mechanisms were unclear. The results of RNE suggest new possibilities for natural products as a sleep-improving supplement.

This study had several limitations. The effect of RNE was tested in a pilot study with a small number of participants. The dropout rate was high as the participants who suffered from disturbances every day readily declined to continue in the study. Some of the measures in the per-protocol analysis, such as sleep disturbance and habitual SE in the PSQI, showed a statistical significance. If the intervention did not quickly show efficacy, the participants were likely to decline further participation after the first dose. In contrast, a significant change was observed among the participants who persisted in the study. As the PSQI is designed to evaluate the status of sleep in the previous 4 weeks, this feature may have interfered with the suggested explanation of the study outcomes.

## 5. Conclusions

A dose of 750 mg/day of RNE improved sleep quality in individuals with subthreshold insomnia. It significantly changed the total ISI and PSQI scores and components over time and showed improvements in WASO and SE. In addition, RNE improved physical and mental health. Clinically significant adverse events and safety-related problems were not observed. These results suggest that RNE may be considered as a preventive or therapeutic agent to improve sleep disturbances. Further clinical trials on the long-term efficacy and safety of RNE administration are warranted.

## Figures and Tables

**Table 1:** Sociodemographic characteristics and sleep status of the included participants at screening (n = 20).

Parameter	
Age (years)	33.40 ± 14.27
19 ≤ age < 30	11 (55.0%)
30 ≤ age < 40	4 (20.0%)
40 ≤ age < 50	1 (5.0%)
50 ≤ age < 60	2 (10.0%)
60 ≤ age < 65	2 (10.0%)
Sex	
Female	14 (70.0%)
Male	6 (30.0%)
Education status (years)	15.95 ± 1.39
High school	10 (50.0%)
≥College	10 (50.0%)
Height (cm)	162.56 ± 8.41
Weight (kg)	60.52 ± 13.50
BMI	22.81 ± 4.28
Social drinking	12 (60.0%)
Smoking	4 (20.0%)
Drinking coffee	15 (75.0%)
Coffee (cups/day)	1.01 ± 0.80
ISI	12.85 ± 1.39
Difficulties in initiating sleep	20 (100.0%)
Frequency (days/week)	4.63 ± 1.79
Difficulties in maintaining sleep	12 (60.0%)
Frequency (days/week)	4.67 ± 2.76
Early morning awakening	11 (55.0%)
Frequency (days/week)	2.92 ± 2.22
Day-time sleepiness	17 (85.0%)
Frequency (days/week)	4.75 ± 2.01
Day-time symptoms	20 (100.0%)
Treatment for insomnia	4 (20.0%)
Medication	4 (20.0%)
Cognitive behavioral therapy	2 (10.0%)
Alcohol and substance abuse	0 (0.0%)

Notes: Values are means ± SD or n (%) of subjects.

**Table 2:** Comparison of Fatigue Severity Scale (FSS) scores for every visit after oral administration of a mixture of *Rhodiola rosea* and *Nelumbo nucifera* extracts (named RNE) (n = 20).

	Visit 1	Visit 2	Visit 3	*p*-Values
FSS	37.65 ± 8.67	38.50 ± 9.24	37.15 ± 7.29	X^2^ = 2.5769, *p* = 0.2757 *
				V1 vs. V2: Z = −0.5611, *p* = 0.5747 † V2 vs. V3: Z = −1.4236, *p* = 0.1546 † V1 vs. V3: Z = −0.3499, *p* = 0.7264 †

Notes: Values are means ± SD. ITT analysis. * Statistical significance evaluated using the Friedman test. † Statistical significance assessed using the Wilcoxon signed-rank test. Abbreviations: V: visit.

## Data Availability

The datasets used and/or analyzed during the current study are available from the corresponding author on reasonable request.

## Supplementary Materials

The following supporting information can be downloaded at: https://www.mdpi.com/article/10.3390/nu16121867/s1, The material includes Tables S1–S4, which depict detailed data, including the sociodemographic characteristics and sleep status at screening, the ISI and PSQI indices, and the FSS and SF-36 for the three visits following the administration of RNE to the 13 participants who completed the trial (per-protocol analysis). The statistical analysis is depicted for the parameters included in the study.
