# Supplementary material for: Mixture of Rhodiola rosea and Nelumbo nucifera Extracts Ameliorates Sleep Quality of Adults with Sleep Disturbance
Source: Nutrients. 2024 Jun 14;16(12):1867. doi: 10.3390/nu16121867 (PMC11206944; doi:10.3390/nu16121867)
Supplement: Supplementary file 1 [file nutrients-16-01867-s001.zip › nutrients-3026912-supplementary.pdf]

Table S1. Sociodemographic characteristics and sleep status at screening of the participants who completed the trial period (N = 13)

Parameter	
Age	34.46 ± 14.82
19 ≤ age < 30	7 (53.8%)
30 ≤ age < 40	2 (15.4%)
40 ≤ age < 50	1 (7.7%)
50 ≤ age < 60	2 (15.4%)
60 ≤ age < 65	1 (7.7%)
Sex	
Female	9 (69.2%)
Male	4 (30.8%)
Education status (years)	15.85 ± 1.34
High school	6 (46.2%)
≥ College	7 (53.8%)
Height (cm)	161.72 ± 9.65
Weight (kg)	61.45 ± 15.14
BMI	23.38 ± 5.00
Drinking	8 (61.5%)
Smoking	3 (23.1%)
Drinking Coffee	11 (84.6%)
Coffee (cups/day)	1.09 ± 0.77
ISI	12.77 ± 1.42
Difficulties in initiating sleep	13 (100.0%)
Frequency (days/week)	4.83 ± 2.03
Difficulties in maintaining sleep	8 (61.5%)
Frequency (days/week)	5.00 ± 2.54
Early morning awakening	7 (53.8%)
Frequency (days/week)	2.43 ± 2.30
Daytime sleepiness	10 (76.9%)
Frequency (days/week)	5.39 ± 1.87
Daytime symptoms	13 (100.0%)
Treatment for insomnia	2 (15.4%)
Medication	2 (15.4%)
Cognitive behavioral therapy	1 (7.7%)
Alcohol and substance abuse	0 (0%)

Notes: Values are the mean ± SD or n (%) of the number of participants.

Table S2. Comparison of Insomnia Severity Index (ISI) and Pittsburgh Sleep Quality Index (PSQI) at every visit after oral administration of a mixture of *Rhodiola rosea* and *Nelumbo nucifera* extract (RNE), PP (N = 13)

	Visit 1	Visit 2	Visit 3	p-values
ISI	12.69 ± 1.38	9.31 ± 3.88	8.15 ± 4.79	X^2 = 10.7234, p = 0.0047*
				V1 vs. V2) Z = -2.3177, p = 0.0205†
				V2 vs. V3) Z = -1.2791, p = 0.2009†
				V1 vs. V3) Z = -2.4730, p = 0.0134†
Global PSQI score	10.62 ± 2.57	8.23 ± 3.35	7.46 ± 3.13	X^2 = 7.5814, p = 0.0226*
				V1 vs. V2) Z = -2.4095, p = 0.0160†
				V2 vs. V3) Z = -1.3171, p = 0.1878†
				V1 vs. V3) Z = -2.6713, p = 0.0076†
Component 1	2.08 ± 0.49	1.54 ± 0.66	1.23 ± 0.44	X^2 = 15.2000, p = 0.0005*
				V1 vs. V2) Z = -2.3333, p = 0.0196†
				V2 vs. V3) Z = -2.0000, p = 0.0455†
				V1 vs. V3) Z = -3.0509, p = 0.0023†
Component 2	2.62 ± 0.77	2.15 ± 0.99	2.00 ± 0.91	X^2 = 5.2667, p = 0.0718*
				V1 vs. V2) Z = -1.7304, p = 0.0836†
				V2 vs. V3) Z = -0.6325, p = 0.5271†
				V1 vs. V3) Z = -1.6513, p = 0.0987†
Component 3	2.00 ± 1.00	1.62 ± 1.12	1.77 ± 1.01	X^2 = 1.1053, p = 0.5754*
				V1 vs. V2) Z = -1.0954, p = 0.2733†
				V2 vs. V3) Z = -0.6325, p = 0.5271†
				V1 vs. V3) Z = -0.5758, p = 0.5648†
Component 4	1.31 ± 1.32	0.77 ± 1.09	0.54 ± 0.78	X^2 = 6.0000, p = 0.0498*
				V1 vs. V2) Z = -1.5521, p = 0.1206†
				V2 vs. V3) Z = -1.3416, p = 0.1797†
				V1 vs. V3) Z = -2.2323, p = 0.0256†
Component 5	1.15 ± 0.38	1.08 ± 0.28	1.00 ± 0.41	F = 0.7347, p = 0.4478*
				X^2 = 1.5000, p = 0.4724*
				V1 vs. V2) Z = -1.0000, p = 0.3173†
				V2 vs. V3) Z = -0.5774, p = 0.5637†
				V1 vs. V3) Z = -1.0000, p = 0.3173†
Component 6	0.15 ± 0.38	0.00 ± 0.00	0.00 ± 0.00	X^2 = 4.0000, p = 0.1353*
				V1 vs. V2) Z = -1.4142, p = 0.1573†
				V2 vs. V3) Z = 0.0000, p = 1.0000†
				V1 vs. V3) Z = -1.4142, p = 0.1573†
Component 7	1.31 ± 0.95	1.08 ± 0.76	0.92 ± 0.76	X^2 = 6.6154, p = 0.0366*
				V1 vs. V2) Z = -1.7321, p = 0.0833†
				V2 vs. V3) Z = -1.4142, p = 0.1573†
				V1 vs. V3) Z = -1.8898, p = 0.0588†

Notes: * Statistical significance as evaluated using the Friedman test.

† Statistical significance as evaluated using the Wilcoxon signed-rank test.

Abbreviations: PP, per-protocol; V, visit

Table S3. Comparison of Fatigue Severity Scale (FSS) at every visit after oral administration of a mixture of *Rhodiola rosea* and *Nelumbo nucifera* extract (RNE), PP (N = 13)

	Visit 1	Visit 2	Visit 3	p-values
Mean ± SD	37.62 ± 10.00	38.54 ± 10.32	36.46 ± 7.36	$X^2 = 2.9130$, p = 0.2330*
				V1 vs. V2) Z = -0.2674, p = 0.7892†
				V2 vs. V3) Z = -1.4236, p = 0.1546†
				V1 vs. V3) Z = -0.6230, p = 0.5333†

Notes: * Statistical significance as evaluated using the Friedman test.

† Statistical significance as evaluated using the Wilcoxon signed-rank test.

Abbreviations: PP, per-protocol; V, visit

Table S4. Comparison of Short Form (36) Health Survey (SF-36) at every visit after oral administration of a mixture of *Rhodiola rosea* and *Nelumbo nucifera* extract (RNE), PP (N = 13)

	Visit 1	Visit 2	Visit 3	p-values
PF	82.56 ± 15.09	85.38 ± 13.76	86.54 ± 11.44	X^2 = 0.9143, p = 0.6331*
				V1 vs. V2) Z = -1.0433, p = 0.2968†
				V2 vs. V3) Z = -0.6325, p = 0.5271†
				V1 vs. V3) Z = -1.2539, p = 0.2099†
RP	59.62 ± 36.14	67.31 ± 25.79	75.00 ± 25.00	X^2 = 3.9200, p = 0.1409*
				V1 vs. V2) Z = -1.1896, p = 0.2342†
				V2 vs. V3) Z = -1.1339, p = 0.2568†
				V1 vs. V3) Z = -1.9944, p = 0.0461†
BP	68.23 ± 18.44	74.46 ± 20.69	75.15 ± 16.98	X^2 = 4.5185, p = 0.1044*
				V1 vs. V2) Z = -1.5724, p = 0.1159†
				V2 vs. V3) Z = -0.1054, p = 0.9161†
				V1 vs. V3) Z = -1.7780, p = 0.0754†
GH	47.77 ± 14.00	55.31 ± 16.92	55.31 ± 13.59	X^2 = 4.0976, p = 0.1289*
				V1 vs. V2) Z = -1.4967, p = 0.1345†
				V2 vs. V3) Z = -0.1799, p = 0.8572†
				V1 vs. V3) Z = -1.9767, p = 0.0481†
VT	42.59 ± 21.37	43.85 ± 18.61	47.69 ± 19.22	X^2 = 1.5122, p = 0.4695*
				V1 vs. V2) Z = -0.3563, p = 0.7216†
				V2 vs. V3) Z = -1.5016, p = 0.1332†
				V1 vs. V3) Z = -0.8213, p = 0.4115†
SF	71.15 ± 21.88	77.88 ± 21.14	75.96 ± 23.64	X^2 = 3.2500, p = 0.1969*
				V1 vs. V2) Z = -1.8226, p = 0.0684†
				V2 vs. V3) Z = -0.3562, p = 0.7217†
				V1 vs.V3) Z = -1.1272, p = 0.2597†
RE	56.41 ± 36.98	76.92 ± 34.39	79.49 ± 32.03	X^2 = 7.4483, p = 0.0241*
				V1 vs. V2) Z = -2.2323, p = 0.0256†
				V2 vs. V3) Z = -0.4252, p = 0.6707†
				V1 vs. V3) Z = -2.2087, p = 0.0272†
MH	62.77 ± 15.61	67.69 ± 14.28	64.00 ± 14.24	X^2 = 4.5714, p = 0.1017*
				V1 vs. V2) Z = -2.1913, p = 0.0284†
				V2 vs. V3) Z = -1.1486, p = 0.2507†
				V1 vs. V3) Z = -0.4352, p = 0.6635†

Notes: * Statistical significance as evaluated using the Friedman test.

† Statistical significance as evaluated using the Wilcoxon signed-rank test.

Abbreviations: PP, per-protocol; V, visit
